# Li-Fraumeni Syndrome, A Rarity Among Rarities: A Case Report and Review of Literature

**DOI:** 10.7759/cureus.45462

**Published:** 2023-09-18

**Authors:** Mariam Elremeli, Philip Idaewor, Noreen Rasheed, Abdalla Saad Abdalla Al-Zawi

**Affiliations:** 1 Paediatrics-Allergy/Immunology, Imperial College London, London, GBR; 2 Histopathology/Cellular Pathology, Mid and South Essex National Health Service (NHS) Foundation Trust, Basildon, GBR; 3 Histopathology/Cellular Pathology, Basildon and Thurrock University Hospital National Health Service (NHS) Foundation Trust, Basildon, GBR; 4 Radiology, Basildon and Thurrock University Hospital National Health Service (NHS) Foundation Trust, Basildon, GBR; 5 General and Breast Surgery, Mid and South Essex National Health Service (NHS) Foundation Trust, Basildon, GBR; 6 General and Breast Surgery, Basildon and Thurrock University Hospital National Health Service (NHS) Foundation Trust, Basildon, GBR; 7 General and Breast Surgery, Anglia Ruskin University, Chelmsford, GBR

**Keywords:** li-fraumeni syndrome, upfront chemotherapy, tp53 mutation, brain pleomorphic xanthoastrocytoma, breast cancer

## Abstract

Li-Fraumeni syndrome (LFS) is a rare inherited cancer susceptibility disorder with a wide tumour spectrum, particularly in children and young adults. Patients with LFS have life-long cancer risk, and the most commonly encountered tumours include soft tissue sarcoma, breast cancer, brain tumours, osteosarcoma, leukaemia and adrenocortical carcinoma. LFS is associated with mutations in the tumour suppressor gene *TP53, *andnearly two-thirds of families with LFS have this germline mutation. However, the diagnosis of LFS is currently based on recognised strict clinical criteria regardless of the genetic mutation status, as a few families with the clinical characteristics and cancer predisposition of LFS do not have *TP53* mutations.

Breast cancer is particularly significant among the common malignancies associated with LFS as it is the most common cancer in women worldwide. We present a case of a 27-year-old woman with unilateral breast cancer, in whom further history revealed a brain tumour at the age of 14 years. Due to the early onset of breast cancer and history of childhood malignancy, we suspected LFS. Genetic testing revealed a *TP53* mutation, further suggesting the diagnosis of LFS. This has important implications in managing this patient's breast cancer, as the need for risk-reducing mastectomy and arranging a special surveillance programme. It also has great implications for the patient’s family members, especially in terms of psychological impact, particularly when the mutation has been detected in children. Also, there is a need for periodic surveillance, which can help in early diagnosis and timely treatment with a more favourable outcome.

## Introduction

Li-Fraumeni syndrome (LFS; OMIM #151623) is a rare, aggressive, dominantly inherited cancer predisposition syndrome with high penetrance and wide clinical variability. Affected individuals could suffer from synchronous or metachronous multiple primary tumours at different sites of the body, and several members of the same family could also be affected [1]. The tumours most frequently associated with LFS are soft tissue sarcomas, breast cancer, brain tumours, osteosarcoma, leukaemia and adrenocortical carcinoma [2]. However, there is great variability in the number of tumours encountered and their clinical behaviour among different patients. Although LFS was first described in 1969 by Frederick Li and Joseph Fraumeni Jr. [3], it was not until 21 years later that the germline mutation of the tumour suppression gene *TP53* was identified as the main genetic defect primarily responsible for this autosomal dominant inherited disease [4]. Nearly two-thirds of individuals with LFS harbour the *TP53* germline mutation, and genetic counselling and *TP53* mutation testing should, therefore, be strongly considered and offered when LFS is suspected. However, because *TP53* mutations are absent in 30% of patients, the diagnosis of LFS is currently based on clinical evaluation and the fulfilment of rigorous criteria independent of genetic mutational status. The Li-Fraumeni-like (LFL) phenotype is designated for patients with incomplete LFS features, and 40% of these patients carry deleterious *TP53* mutations [5]. Infants and young children are increasingly diagnosed with LFS, and the introduction of multi-gene panels and next-generation sequencing (NGS) has enabled researchers and oncologists to recognise the significant role of LFS in the pathogenesis of childhood cancers. Germline pathogenic *TP53* variants were found in 80% of children with rhabdomyosarcoma and diffuse anaplasia [6] as well as in 40% of children with choroid plexus carcinoma [7], and noticeably, there was no clear family history of such a mutation in most of these children [8]. These patients have a high lifetime cumulative cancer risk. The risk of developing a primary malignancy is 50% by the age of 31 years in women and 46 years in men [2]. Properly timed genetic testing and counselling would significantly change the course of the disease and improve management outcomes.

Despite the breast harbouring malignant lesions from remote sites, primary breast cancer is the most common cancer in women worldwide. According to GLOBOCAN data, it accounted for 25.4% of the total number of new cases of all cancers diagnosed in 2018 [9-14]. Moreover, breast cancer is one of the most prominent primary tumours in LFS, accounting for up to 80% of all cancer cases in females with LFS between the ages of 16 and 45 years. It has been agreed that the onset of breast cancer before the age of 40 years is an operational diagnostic criterion that should prompt clinicians to consider genetic testing for cancer-related mutations, as recent studies suggest that 2.2% to 7.7% of women younger than 31 years old with breast cancer have a *TP53* pathological variant [15]. Herein, we present the case of a young woman with early-onset breast cancer and a history of brain tumours as a child, prompting the diagnosis of LFS.

## Case presentation

**Figure 1:**
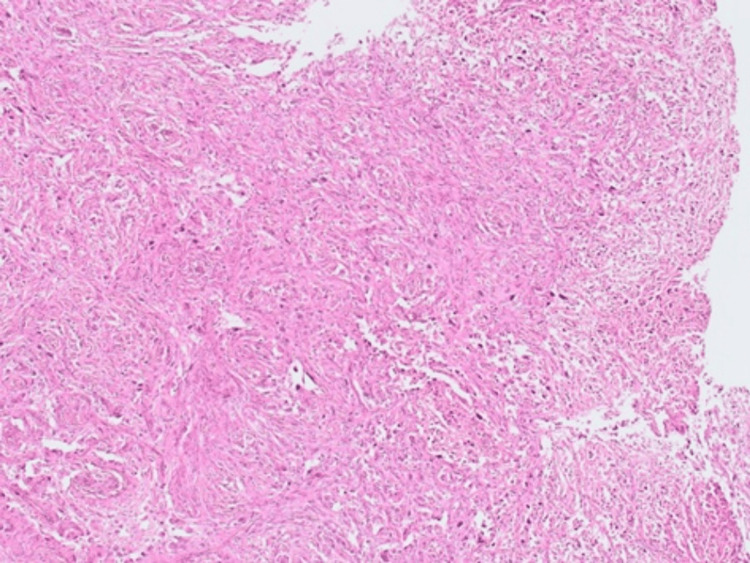
The brain tumour (pleomorphic xanthoastrocytoma) is composed of very pleomorphic hyperchromatic cells with abundant eosinophilic cytoplasm. Some cells show intracytoplasmic vacuolation and some multinucleate. Mitosis is not prominent and no necrosis is seen (×40).

**Figure 2:**
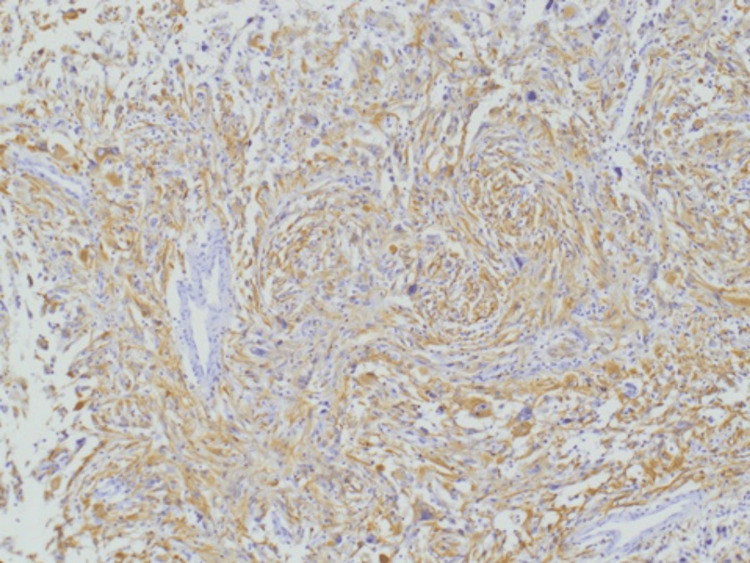
Brain pleomorphic xanthoastrocytoma with diffuse immunoreactivity for GFAP (×10). GFAP, glial fibrillary acidic protein

A 27-year-old female patient presented with a short history of a painful left breast mass and bilateral clear nipple discharge. Past medical history revealed a brain tumour at the age of 14 years, which was treated with a craniotomy and surgical excision. Histology of the excised brain tumour at the time showed benign pleomorphic xanthoastrocytoma (Figures 1-2), and the patient received follow-up care for five years before she was discharged from oncology care.

**Figure 3:**
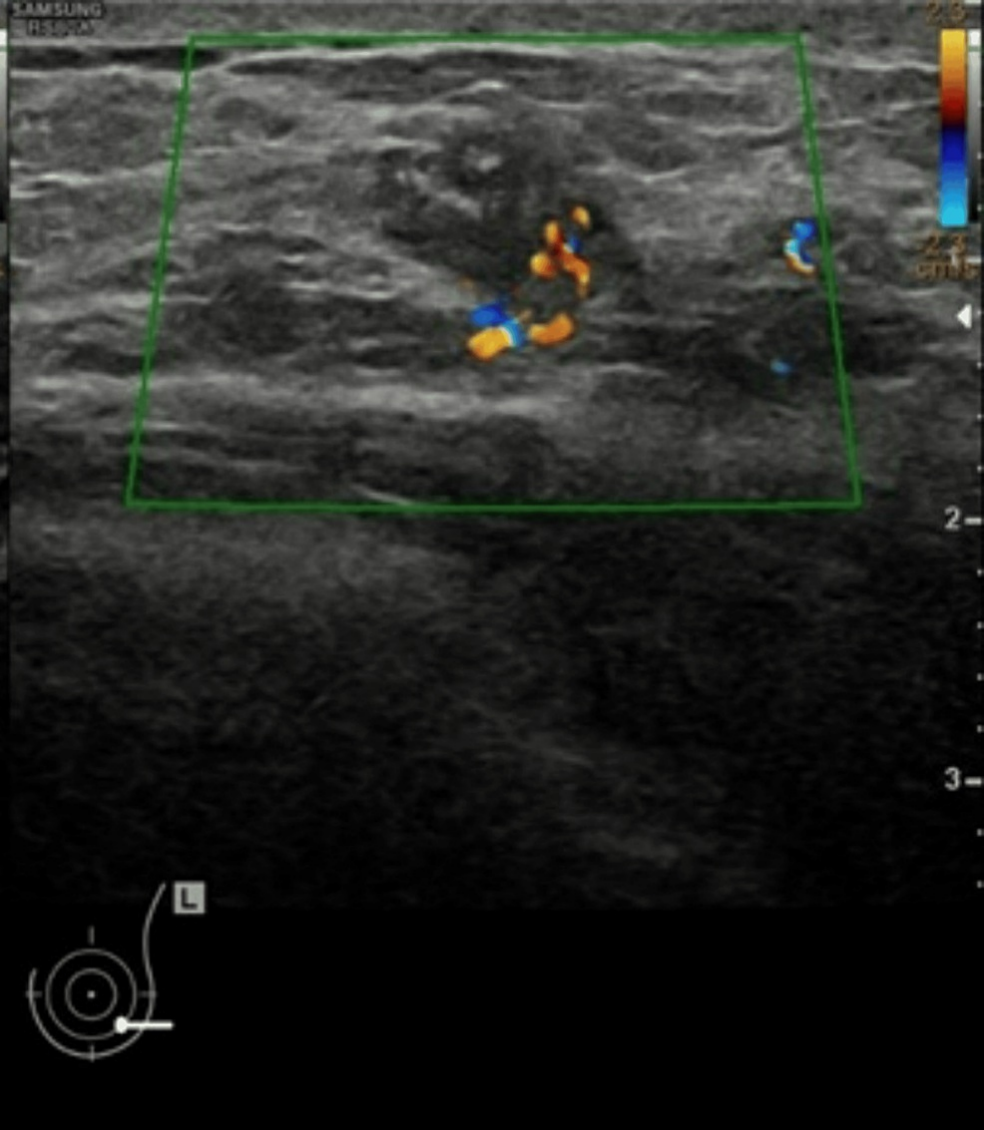
Left breast ultrasound scan revealed a 19-mm irregular suspicious lesion in the lower outer quadrant with increased vascularity (green trapezoid).

She had menarche at the age of 14 years and one pregnancy at 25 years of age, and she has a three-year-old daughter. The patient is a smoker and is currently not using any medications; however, she has previously used oral contraceptive pills. She has neither a family history of breast or ovarian cancer nor a history of other malignancies or unexplained early deaths in the family. On physical examination, no lumps could be identified in the right breast on palpation. By contrast, a poorly defined, firm mass was noted in the inner lower quadrant of the left breast, measuring approximately 2 cm, and no axillary or cervical lymphadenopathy was detected. A left breast ultrasound scan revealed a 1.9 cm irregular suspicious lesion in the lower outer quadrant with increased vascularity (Figure 3). 

**Figure 4:**
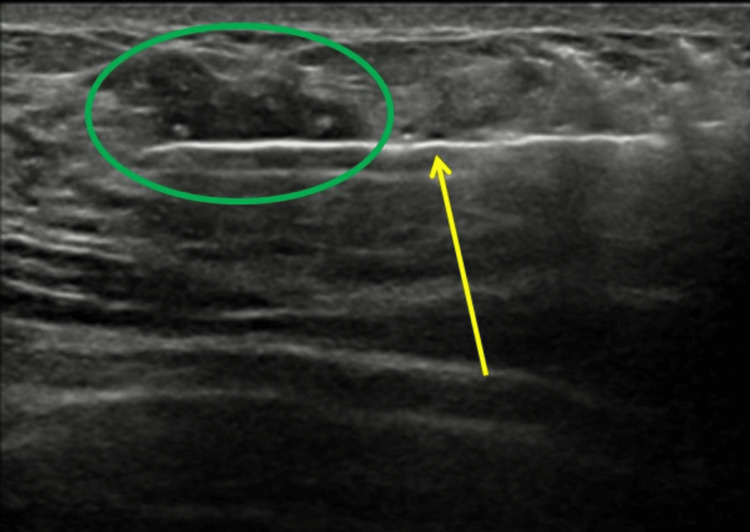
The suspicious irregular lesion within the left breast's lower inner quadrant (green circle) was targeted with an ultrasound-guided core biopsy needle (yellow arrow).

The lesion was targeted for ultrasound-guided core needle biopsy (Figure 4).

**Figure 5:**
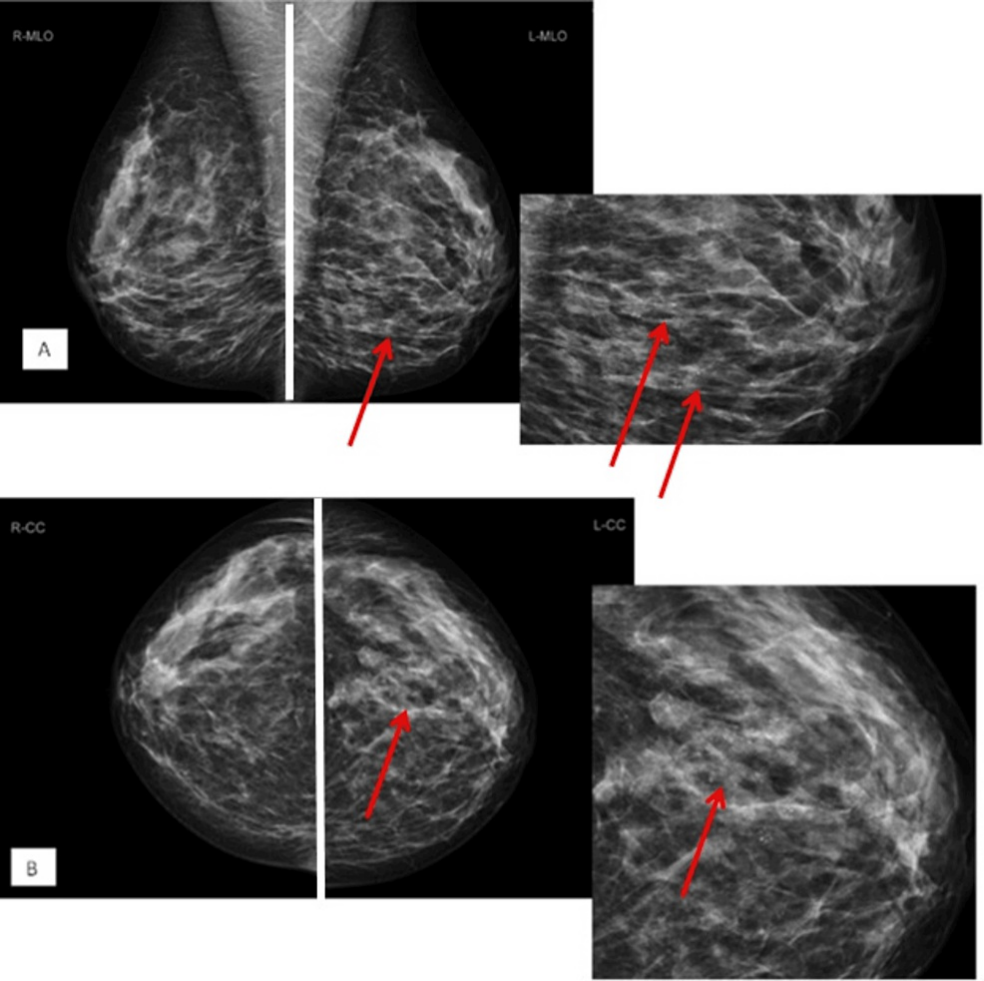
Bilateral mammograms with (A) MLO view and (B) CC view demonstrate heterogeneously dense fibroglandular breast parenchymal tissues. Within the lower quadrant on the MLO view and the middle part on the CC view on the left side, there is a large area of suspicious cluster of pleomorphic micro-calcifications. No definite associated discrete mass was identified. Appearances are highly suspicious of malignancy. MLO, mediolateral oblique; CC, craniocaudal

**Figure 6:**
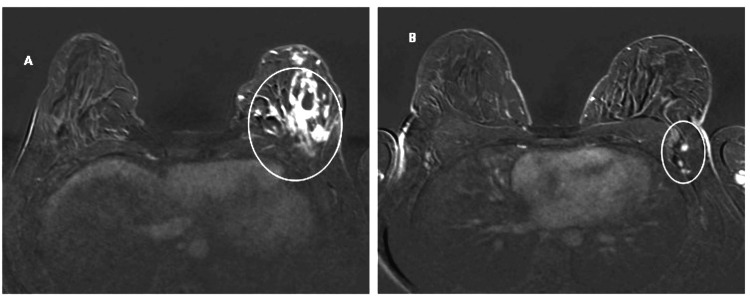
(A) Within the left breast, conglomerate abnormal enhancements are seen in the lower half, measuring 9.8 cm in anteroposterior, 6.7 cm in mediolateral and 3.5 cm in craniocaudal dimensions (indicated by the white circle), highly suggestive of a large malignancy; (B) a small although slightly prominent node in the left axillary tail is seen, as indicated by a white circle.

Mammography demonstrated a lesion with high suspicion of cancer, with a larger area of micro-calcification extending up to 100 mm in the maximum measurement (Figure 5), and the MRI scan showed the involvement of the axillary tail of the left breast (Figure 6).

**Figure 7:**
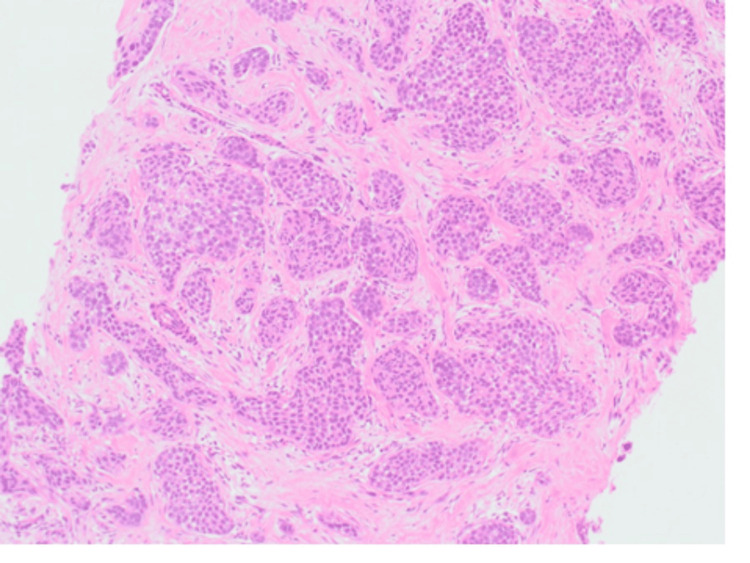
Left breast core biopsy shows grade II invasive carcinoma, NST (×10). NST, no special type

**Figure 8:**
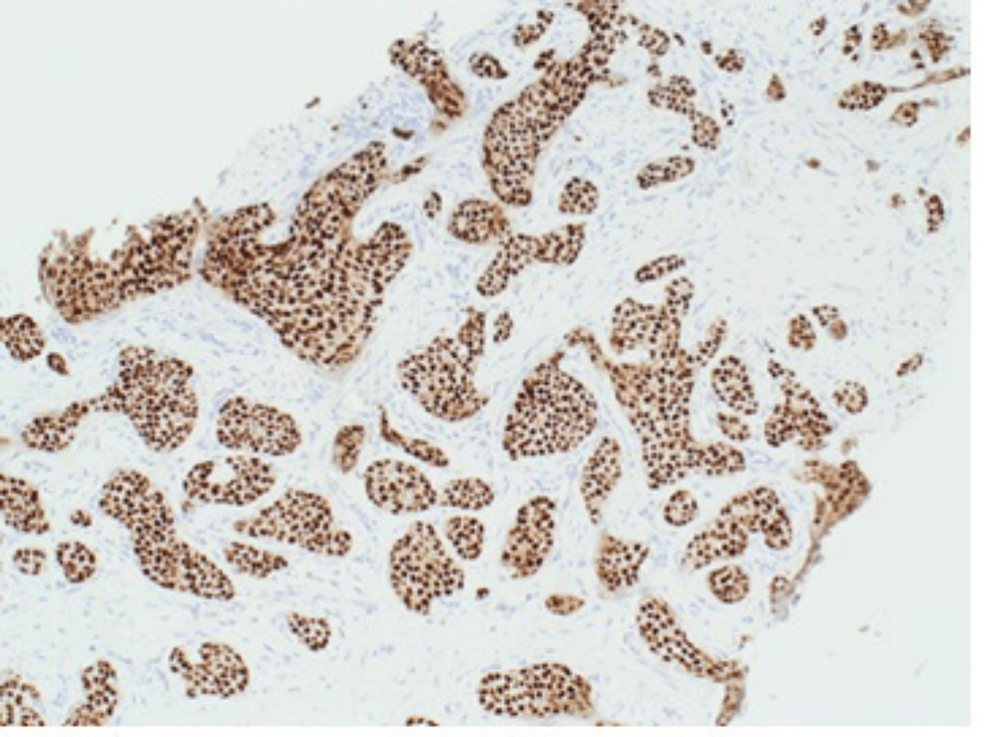
Left breast core biopsy shows grade II, invasive carcinoma cells with strong expression of ER (×10). ER, oestrogen receptor

Histological examination of the ultrasound-guided core needle biopsy revealed grade 2 invasive ductal carcinoma (IDC) of no special type (Figure 7). It was both oestrogen receptor (ER) positive (Figure 8) and progesterone receptor (PR) positive, and it had a Ki-67 index of 8%. Human epidermal growth factor receptor 2 (HER2) was borderline; however, the fluorescence in situ hybridisation (FISH) test showed amplification.

**Figure 9:**
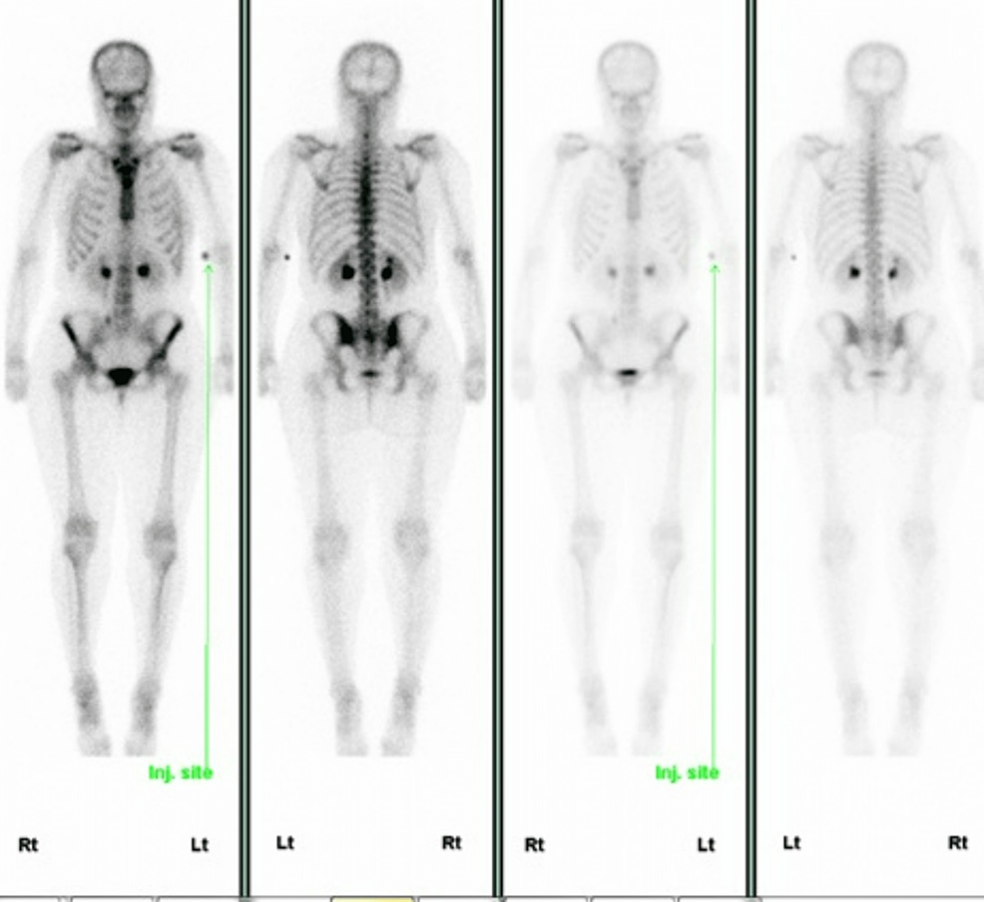
Whole-body bone scan showed no bony metastasis.

Screening for metastatic disease by computed tomography and whole-body bone scan (Figure 9) showed no evidence of regional lymph node, pulmonary, bone or hepatic metastases.

The breast multidisciplinary care team recommended staging a sentinel lymph node biopsy, which revealed one out of three with a micro-metastatic focus. The final pre-treatment staging was pT3N1 (micro)M0. Genetic testing was carried out, and no BRCA1, BRCA2 or PALB2 mutations were detected; however, the analysis of germline *TP53* detected the pathogenic mutation TP53 c.844C > T (p.Arg282Trp) (NM_000546). The analysis was performed in peripheral blood by polymerase chain reaction (PCR) and Sanger sequencing, and when the fraction of the pathogenic variant was approximately 50%, the diagnosis of LFS was established. A whole-body MRI scan was performed to screen for other primary or secondary malignancies, typically associated with LFS, and revealed no other abnormalities. Given the bilateral nipple discharge that the patient presented with and the history of brain tumours, hyperprolactinemia was suspected; however, the serum prolactin level was within normal limits, and the brain MRI showed no pituitary microadenoma. Upfront chemotherapy and HER2 blockade were suggested and initiated. The patient completed four cycles of docetaxel, pertuzumab and trastuzumab, followed by an additional four cycles of epirubicin with cyclophosphamide. After chemotherapy, the patient underwent a left mastectomy with immediate deep inferior epigastric perforator (DIEP) reconstruction with the intention of a delayed right breast risk-reducing mastectomy. The postoperative histology was consistent with a complete pathological response to treatment. HER2 blockade continued after surgery in addition to tamoxifen (planned for 10 years). However, the patient declined the last three doses of trastuzumab as well as further tamoxifen therapy due to associated side effects. The patient is currently well, without any signs or symptoms of cancer recurrence. The patient’s three-year-old daughter underwent genetic testing, and there was no evidence of a *TP53* mutation. The familial nature of the disease and the importance of early detection were discussed with other family members, and genetic testing was offered. Genetic tests of both patient’s parents showed no evidence of *TP53 *mutation. The patient remains under lifelong surveillance with an annual whole-body MRI.

## Discussion

Approximately 10% of women with newly diagnosed breast cancer have a pathogenic variant mutation in the cancer susceptibility gene [16], and the presence of familial aggregation, early age of onset and multiple primary malignancies should prompt evaluation for familial cancer syndromes in patients with breast cancer. Hereditary breast and ovarian cancer syndrome is the most common familial cancer syndrome and is most often associated with pathogenic variant mutations in BRCA1 or BRCA2, which were both negative in our patient. The rare LFS, however, is another familial cancer syndrome associated with the *TP53* mutation; carriers have a very high lifetime risk of developing cancer. The overall cumulative cancer incidence is estimated to be 73% to 100% by the age of 70 years [17]. Patients with LFS who carry the germline mutation in *TP53* and develop breast cancer typically present with a very early age of onset, have an aggressive form of the disease and have been reported to show resistance to treatment, in addition to the inherited ongoing risk of multiple primary and radiation-induced secondary tumours [18-20]. Furthermore, most women with breast cancer and LFS have HER2-positive breast tumours. In our patient, the HER2 test was borderline; however, the FISH test was amplified. HER2-positive breast cancer is aggressive; however, therapies directed at HER2 receptors have favourably changed the outcome. The chemotherapy regimen choice for our patient was based on her fitness, tumour size and HER2 receptor expression status. The patient exhibited a good response to upfront chemotherapy and dual HER2 blockade, and postoperative histology was consistent with a complete pathological response to treatment. Routine genetic mutation screening has been recommended for all women who develop breast cancer before the age of 40, regardless of their family history [15]. The detection of germline mutations in this high-penetrance cancer susceptibility gene has extremely significant implications for patient management and often leads to the adoption of preventive measures such as risk-reducing mastectomy and avoiding excessive radiation exposure during the screening or surveillance period. Many authors recommend the use of breast and whole-body MRI as an imaging follow-up modality [18-19]. Despite some authors’ advice against radiotherapy after breast cancer surgery, as it may induce oncogenesis [19], Hendrickson et al. presented a series of 14 patients who received radiotherapy with curative intent as part of their breast cancer management. The authors found that four out of 14 patients treated with radiotherapy had developed in-field malignancies. All these tumours had the same histology as the primary breast cancer, suggesting local recurrences rather than radiotherapy-induced malignancies. The authors recommend that adjuvant radiotherapy be considered as part of the treatment algorithm if clinically indicated [2]. Furthermore, a detected *TP53* mutation has important implications for a patient’s relatives, who should be offered genetic testing for the same mutation. The UK Cancer Genetics Group (UKCGG) has recommended that surveillance protocols based on international guidelines be offered to *TP53* carriers from birth. Individuals with LFS have a high lifetime cancer risk that approaches 100% by the age of 70 years. However, more than half of all tumours occur before the age of 30 years [20]. In fact, 50% of patients with a malignant tumour develop a second tumour over the next 10 years [13,20]. Our patient, however, developed two primary tumours at different sites of the body within 12 years. The two factors highly suggested the LFS diagnosis, namely, the metachronous primary tumours and the very early age of onset of breast cancer. The detailed, cancer-specific family history over the last three generations could not identify any involvement of cancer or cancer syndromes in family members. Brain tumours can also be associated with LFS, and approximately half of choroid plexus carcinomas arise in the setting of LFS due to germline *TP53* mutations. It has recently been recommended that genetic counselling and germline sequencing be routinely offered to all children with brain tumours, as recognition of germline mutations allows for appropriate surveillance and screening and leads to early detection and potentially life-saving intervention. Our patient had a history of brain tumours and craniotomies as a child. Histology of the excised brain tumour showed pleomorphic xanthoastrocytoma (Figures 1-2), a benign form of glioma with a favourable prognosis. The implication of the genetic results was to offer the patient risk-reducing contralateral mastectomy and annual whole-body MRI surveillance. The current recommendation is to avoid unnecessary radiation exposure in patients with LFS due to the high risk of radiation-related malignancies [18-19]. The published research has revealed that the frequency of de novo mutations in *TP53* is estimated to be approximately 25% [18]. Our patient’s parents and her three-year-old daughter underwent genetic testing, and no evidence of *TP53* mutation was found. Moreover, no family history of malignancy was detected, which could well imply that our patient had a de novo mutation. Genetic testing and screening have been associated with increased distress and anxiety in individuals suspected of familial cancer syndrome, and appropriate psychological support and genetic counselling should be provided to these individuals before and after genetic testing [17]. More evidence is accumulating regarding the importance of intensive surveillance and screening in children and adults with LFS, as early tumour detection improves their survival rate. The expert international panel on LFS recommends that all patients diagnosed with LFS be offered cancer surveillance as soon as the clinical or molecular LFS diagnosis is established and that the modified *Toronto protocol* surveillance guidelines be followed [17-18]. However, the wide clinical variability of LFS makes the development of an effective surveillance program challenging. Regular breast examinations and breast and whole-body MRI scans are the recommended screening tests, and they were performed for our patient as a surveillance modality.

## Conclusions

Patients with LFS have a lifelong cancer risk, and the absence of a family history of cancer does not exclude the presence of this aggressive tumour predisposition syndrome, which can arise due to an acquired de novo germline mutation. It is especially important to keep a high index of suspicion for the diagnosis of LFS in patients younger than 18 years of age with brain tumours and in patients with breast cancer aged <40 years, as early detection among patients with genetic mutations enables modification of the course of the disease, optimisation of treatment and prevention of further malignancies through the development and implementation of extensive, risk-adapted cancer surveillance programmes.
